# Molecular Circuits of Resolution in Airway Inflammation

**DOI:** 10.1100/tsw.2010.143

**Published:** 2010-07-07

**Authors:** Troy Carlo, Bruce D. Levy

**Affiliations:** Pulmonary and Critical Care Medicine, Brigham and Women's Hospital and Harvard Medical School, Boston, MA, USA

**Keywords:** resolution, anti-inflammatory, polyunsaturated fatty acid, mediator, lipoxin, resolvin, protectin, airway inflammation, asthma, acute lung injury, acute respiratory distress syndrome

## Abstract

Inflammatory diseases of the lung are common, cause significant morbidity, and can be refractory to therapy. Airway responses to injury, noxious stimuli, or microbes lead to leukocyte recruitment for host defense. As leukocytes respond, they interact with lung resident cells and can elaborate specific mediators that are enzymatically generated from polyunsaturated fatty acids via transcellular biosynthesis. These bioactive, lipid-derived, small molecules serve as agonists at specific receptors and are rapidly inactivated in the local environment. This review will focus on the biosynthesis, receptors, cellular responses, and *in vivo* actions of lipoxins, resolvins, and protectins as exemplary molecular signaling circuits in the airway that are anti-inflammatory and proresolving.

### INTRODUCTION

Airway inflammation is part of a coordinated host response to infection, injury, or other noxious stimuli and is fundamental to host defense[2]. This process is so common that it is experienced by most individuals frequently throughout their life, such as during a simple community-acquired bronchitis. The natural course of mild airway inflammation is to resolve entirely as the irritation or infection abates[3]. In response to certain stimuli, airway inflammation can be so robust as to lead to bystander tissue injury and contribute to pathophysiology[1]. For example, overexuberant airway inflammation can lead to the acute respiratory distress syndrome (ARDS), a pathologic condition of severe inflammation that is life threatening[4]. In some respiratory conditions, acute inflammation can convert to chronic inflammation, usually with the recruitment of the adaptive immune system, and chronic airway inflammation is part of the pathogenesis of many common lung diseases[2], including asthma[5], which impacts as many as one in 15 adults in the U.S.[6]. The cellular effectors of acute and chronic airway inflammation are evident in sputum, bronchoalveolar lavage fluid (BALF), and lung histology. In general, airway inflammation in acute inflammatory diseases is comprised of cellular effectors that are distinct from those of chronic responses. Acute inflammation, as in acute lung injury (ALI) or ARDS, recruits and activates neutrophils (PMNs)[4]. As this inflammation resolves, PMNs undergo apoptosis[7] and are cleared by macrophages[8]. Allergic airway inflammation consists of eosinophils (EOS) and effector T lymphocytes with modulatory roles for inflammatory macrophages, mast cells, dendritic cells, and structural cells[9].

The molecular signals that initiate acute and provoke chronic inflammation have been the subject of extensive investigation and include cytokines, chemokines, and select lipid mediators. More recently, several anti-inflammatory molecular circuits that also actively promote resolution of tissue inflammation have been uncovered, including the identification of natural small molecules derived from polyunsaturated fatty acids (PUFAs) that are part of a new genus of anti-inflammatory and proresolving mediators (reviewed in [10]). The lipoxins (LXs), resolvins, and protectins are three families of chemical mediators in this genus that are now appreciated to promote the resolution of lung inflammation and they will be the focus of this review on the molecular circuits of resolution of airway inflammation. Information will also be provided on their relationship to both physiologic catabasis and the pathobiology of select lung diseases.

### LIPOXINS

#### Biosynthesis

Lipoxins (LXs) are lipoxygenase (LO)–derived products of arachidonic acid (AA, C20:4) that are predominately generated during cell-cell interactions at sites of vascular or tissue inflammation (reviewed in [11,12]). At sites of inflammation or injury in the lung vasculature, LX formation can occur when platelets interact with activated leukocytes that generate 5-LO–derived leukotriene $A_4$ ($LTA_4$) from AA. Platelet 12-LO, acting as a LX synthase, can then convert $LTA_4$ to LXs. In lung parenchyma, infiltrating leukocytes interact with structural cells to generate LXs via a distinct biosynthetic pathway. In particular, PMN-derived $LTA_4$ can be converted by airway epithelial cell 15-LO to generate LXs (reviewed in [13]). There are additional LX biosynthetic pathways, including the transformation of 15-LO–derived 15-hydroperoxy-eicosatetraenoic acid (15-H(p)ETE) by 5-LO to LXs. 15-epimer-LXs (15-epi-LXs) are also found in respiratory tissues[14]. The 15-epi-LXs are generated by 5-LO–mediated conversion of 15(R)-hydroxy-eicosatetraenoic acid (15(R)-HETE) to 15-epi-$LXA_4$ and 15-epi-$LXB_4$ (reviewed in [11]). Both aspirin-acetylated cyclooxygenase (COX)–2 and cytochrome p450 activities can catalyze the formation of 15(R)-HETE from AA. Of note, statins also demonstrate the ability to trigger 15-epi-$LXA_4$ formation[15,16,17]. Statins and pioglitazone can initiate post-translational modification of COX-2 and 5-LO in rat cardiomyocytes to influence AA conversion to 15-epi-$LXA_4$[15,16]. In addition, cell-cell interactions between PMNs and airway epithelial cells in the presence of statins leads to 15-epi-$LXA_4$ biosynthesis, in which the epoxygenase cytochrome p450 product 14,15-epoxyeicostrienoic acid influences AA metabolism[17].

#### Signaling

$LXA_4$ and 15-epi-$LXA_4$ are both agonists for a $LXA_4$ receptor termed ALX/FPR2, which is a seven-transmembrane-spanning G protein-coupled receptor (GPCR) that binds these ligands with high affinity (reviewed in [18]). The glucocorticoid-induced protein annexin 1 and related peptides can also bind to ALX, although with lower affinity than $LXA_4$[19]. Of interest to lung biology, ALX is expressed on human airway epithelial cells[20] and leukocytes[20,21,22], and can be induced by select inflammatory mediators[23]. LX signaling is not limited to interactions with ALX. LXs can act as antagonists at $CysLT_1$ receptors[24] and can also signal via the aryl hydrocarbon receptor[25].

LXs interact with ALX to evoke cell type–specific responses that are anti-inflammatory and proresolving (Fig. 1). For example, anti-inflammatory actions for LX signaling through ALX include inhibition of PMN and EOS chemotaxis and activation[26,27,28,29], and proresolving actions include increasing macrophage phagocytosis of apoptotic PMNs to clear inflamed tissue[30]. In human leukocytes,

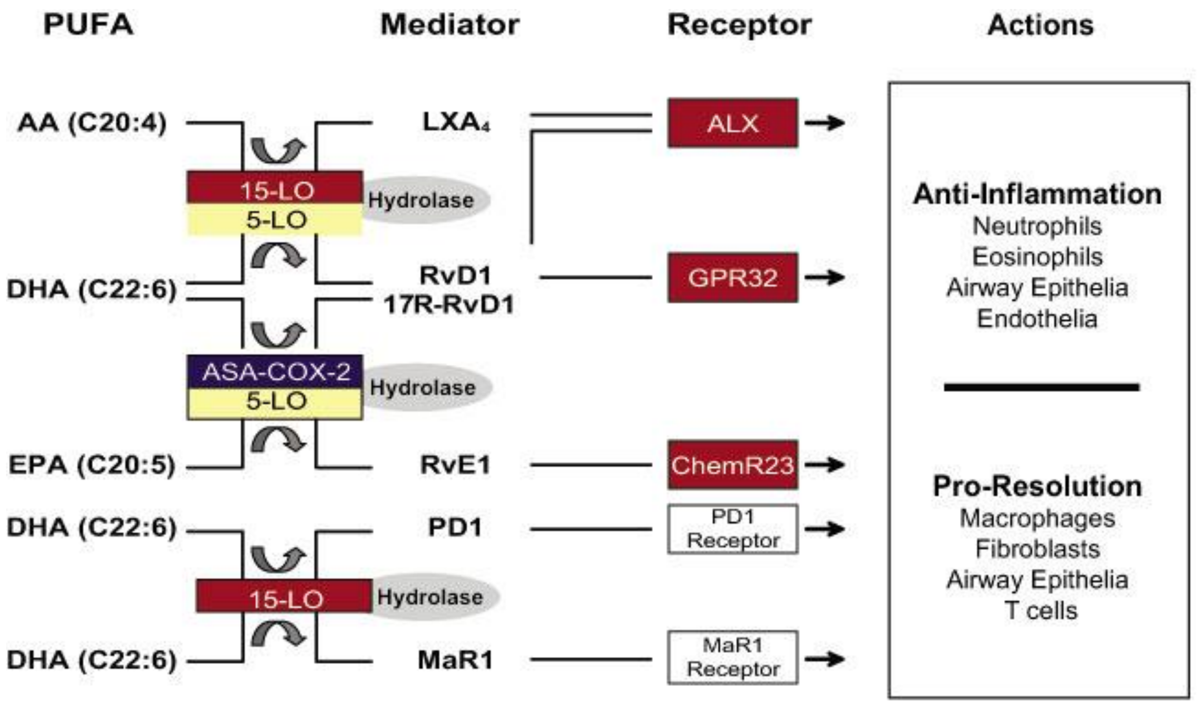

**FIGURE 1.** Molecular circuits of resolution for airway inflammation. During airway inflammation, select PUFAs are enzymatically transformed to bioactive mediators in complex biosynthetic circuits. These mediators elicit pathway- and cell type–specific responses that are both anti-inflammatory and proresolving.

LX-ALX interactions trigger specific intracellular signal events, such as blocking the phosphorylation of leukocyte-specific protein 1 in PMNs and α-fodrin and f-actin in EOS[31,32].

ALX signaling can also regulate the apoptotic fate of PMNs. There are several nonlipid ligands for ALX with potent actions on PMNs. Serum amyloid A (SAA) is an acute-phase reactant that binds to ALX and produces proinflammatory signals that delay PMN apoptosis[33]. In contrast, the glucocorticoid-induced protein annexin 1 and related peptides can bind to ALX and promote PMN apoptosis (reviewed in [18,34]). The lipid and protein ligands bind to distinct ALX domains[18] and 15-epi-$LXA_4$ can block the antiapoptotic effects of SAA[33].

$LXA_4$ also blocks polyisoprenyl phosphate remodeling to regulate cell activation[35]. Presqualene diphosphate (PSDP) is a polyisoprenyl diphosphate present in resting cell membranes that serves as an intracellular stop signal for PMNs. Rapid dephosphorylation of this counter-regulatory signaling molecule can facilitate transient cellular responses to provocative stimuli[36]. The addition of exogenous PSDP, but not its related monophosphate, presqualene monophosphate (PSMP), blocks $O_2$- production from human PMNs. In addition, PSDP and PSDP mimetics that resist inactivation can inhibit important signaling checkpoints for cell activation, namely phosphatidylinositol 3-kinase (PI3K) and phospholipase D (PLD)[35,37,38]. Stable LX analogs dramatically block PMN PSDP remodeling in response to proinflammatory agonists[35]. LX-mediated inhibition of PSDP remodeling is linked to its anti-inflammatory effects on PMN functional responses, including $O_2$- generation[35,38]. Recently, polyisoprenyl diphosphate phosphatase 1 (PDP1) (originally identified as CSS2α and PPAPDC2) was characterized as a pivotal phosphatase for PSDP remodeling to PSMP[39,40] and may serve as a target for LXs to prevent PSDP remodeling. Taken together, LX's role in blocking PSDP remodeling, PSDP's ability to block enzymatic activities critical to PMN activation, and PDP1's ability to convert PSDP to PSMP are highly suggestive of an integrated signal transduction pathway that regulates PMN-mediated inflammation and sets the stage for catabasis. Given LX's cell type–specific actions, it is not surprising that ALX also initiates cell type–specific signaling circuits (reviewed in [18]).

### Cellular Responses to LXs

In health, the ability to regulate leukocyte accumulation and activation in the lung is fundamental to homeostatic responses. LXs can inhibit granulocyte locomotion, shape change, transmigration, and degranulation[28,29,35,41,42]. In contrast, LXs stimulate monocytes and macrophages in a nonphlogistic manner to enhance monocyte adherence, locomotion and transmigration, and macrophage phagocytosis of apoptotic PMNs and microbial products[30,43,44]. Together, these LX-mediated cellular responses are both anti-inflammatory for PMNs and EOS, and proresolving for clearance of inflamed tissue by monocytes and macrophages. In addition to these leukocyte-specific actions, LXs promote restitution of injured respiratory epithelia by stimulating bronchial basal epithelial cell proliferation, inhibit release of proinflammatory cytokines IL-6 and IL-8, and block PMN transmigration across differentiated human bronchial cells[20]. ALX receptor expression in both proximal and distal epithelial cells is increased after injury[20]. Consistent with a role in tissue homeostasis, LXs also block inflammatory angiogenesis and endothelial cell migration in response to proinflammatory mediators[45]; IL-1β–mediated synthesis of IL-6, IL-8, and matrix metalloproteinases by fibroblasts[46]; and leukotriene $E_4$ and IL-13 primed airway smooth muscle migration towards to platelet-derived growth factor[47]. LX's regulatory actions on this broad array of cell types relevant to lung catabatic responses suggests a pivotal role for this family of mediators in lung physiology.

### LXs in Models of Lung Inflammation

To integrate these cellular actions for LXs into more complex settings, the *in vivo* impact of LXs and LX-stable analogs has been investigated in several experimental models of lung disease (Table 1). These compounds have been extensively studied in experimental asthma. In murine models of asthma, animals are systemically sensitized to allergen and subsequently aerosol challenged in order to direct the allergic inflammation to the airway. Administration of LX analogs prior to aerosol challenge potently blocks the development of allergic airway inflammation and airway hyper-responsiveness, decreases EOS and T-cell accumulation, and dampens $T_h2$ cytokine levels[27,48]. Upon cessation of allergen challenge, the allergic airway responses are self-limited and, within 7 days, EOS and T-cell numbers return to near baseline. During this resolution phase, endogenous generation of $LXA_4$ increases[49]. The administration of a LX-stable analog after the final aerosol challenge accelerates resolution by dramatically decreasing lung leukocyte numbers and selectively regulating airway cytokine levels, including IL-17, IL-23, and IL-6[49]. In mice and humans, IL-17 is generated in inflamed lung and associated with chronic inflammatory diseases[50]. In transgenic mice expressing human ALX receptors, allergic airway responses are blocked and development of allergy (as determined by total IgE levels) is markedly reduced[27]. Of additional note, mice deficient in ALX display a proinflammatory phenotype[51]. LX's anti-inflammatory effects are not limited to mouse models of allergic lung inflammation. For example, administration of LX-stable analogs also potently blocks edema and antigen-driven recruitment of PMNs and EOS in a rat model of allergic pleurisy[26]. Taken together, these data support the notion that LX signaling through ALX is a potent molecular circuit for the regulation of allergic inflammation.

In addition to experimental asthma, LX-stable analogs have been used in other models of lung inflammation (Table 1). In a model of pulmonary fibrosis, LXs block bleomycin (BLM)–induced airway inflammation and fibrosis[52,53]. Mice receiving concurrent BLM and LXs, or animals given LXs as a treatment post-BLM exposure, both display decreased cellular infiltration, edema, and collagen deposition in the lung[52]. Moreover, LXs enhance survival from BLM toxicity[52]. In this model, lung collagen deposition is correlated with fibrosis and increased ALX mRNA expression is associated with a decrease in lung collagen[53].

**TABLE 1**
**Effects of Anti-Inflammatory and Proresolving Mediators on Human Lung Disease and Murine Models of Lung Disease**

| Model or Disease State | Species | Compound | Effect | Ref. |
|---|---|---|---|---|
| Asthma | Human | $LXA_4$ | Blocked $LTC_4$-mediated bronchoconstriction | [99] |
| Allergic airway inflammation | Mouse | $LXA_4$/LX analogs (anti-inflammatory) | Decreased EOS and T cells in BALFs and lungs | [27,48] |
| | | | Decreased $T_H2$ cytokine levels in BALFs | |
| | | | Decreased airway hyper-responsiveness | |
| | | LX analogs (proresolving) | Enhanced EOS and T-cell clearance from lungs | [27,48] |
| | | PD1 (anti-inflammatory) | Decreased EOS and T cells in BALFs and lungs | [97] |
| | | | Decreased $T_H2$ cytokine levels in BALFs | |
| | | | Decreased airway hyper-responsiveness | |
| | | | Decreased mucus metaplasia | |
| | | PD1 (proresolving) | Enhanced EOS and T-cell clearance from lungs | [97] |
| | | RvE1 (anti-inflammatory) | Decreased EOS and T-cells in BALFs and lungs | [49,80] |
| | | | Decreased $T_H2$ cytokine levels in BALFs | |
| | | | Decreased airway hyper-responsiveness | |
| | | | Decreased mucus metaplasia | |
| | | RvE1 (proresolving) | Enhanced EOS and T-cell clearance from lungs | [49] |
| | | | Improved $T_H2$ cytokine levels in BALFs | |
| | | | Improved airway hyper-responsiveness | |
| | | | Improved mucus metaplasia | |
| Acid-initiated ALI | Mouse | PSDP mimetic | Decreased lung PMNs | [37] |
| | | Lovastatin | Increased 15-epi-$LXA_4$ in BALFs | [17] |
| | | | Decreased lung PMNs | |
| | | Aspirin | Increased 15-epi-$LXA_4$ in BALFs | [54] |
| | | | Decreased lung PMNs | |
| | | COX-2 inhibitor | Decreased $LXA_4$ in BALFs | [54] |
| | | | Increased lung PMNs | |
| | | RvE1 | Decreased lung PMNs | [81] |
| | | | Decreased select proinflammatory mediators | |
| Carrageenan-induced lung injury | | 15-epi-$LXA_4$ | Decreased lung PMNs | [55] |
| | | | Increased lung macrophages | |
| | | | Promoted PMN apoptosis | |
| *E. coli* peritonitis-associated lung injury | | 15-epi-$LXA_4$ | Decreased lung PMNs | [55] |
| | | | Promoted PMN apoptosis | |
| | | | Decreased mortality | |
| Pneumonia | Mouse | RvE1 | Enhanced bacterial clearance | [81] |
| | | | Decreased lung PMNs | |
| | | | Decreased proinflammatory cytokine levels in BALFs | |
| | | | Decreased mortality | |

$LXA_4$, lipoxin $A_4$; PD1, protectin D1; RvE1, resolvin E1; PSDP, presqualene diphosphate; BALFs, bronchoalveolar lavage fluids.

In a self-limited model of ALI by hydrochloric acid, LXs decrease inflammation and promote resolution[54]. Using a nonlethal model of ALI in which acid is selectively instilled into only one lung allows for investigation of catabatic responses during ALI resolution. Using this model, important roles were uncovered for COX-2 in the timely resolution of ALI, in part via generation of LXs[54]. Intratracheal instillation of carrageenan plus myeloperoxidase produces PMN-mediated lung injury. Mice that received 15-epi-$LXA_4$ treatment 24 h postinjury displayed reductions in PMN numbers, total protein amount, and IL-6 levels in BALFs[55]. Promoting PMN apoptosis is a potent proresolving mechanism[56]. 15-epi-$LXA_4$ decreased lung PMNs by enhancing PMN apoptosis, as measured by cytoplasmic histone-associated DNA fragments and PMN caspase-3 activity[55]. In addition, human ALX transgenic mice are protected from ALI[54]. Statins also facilitate resolution in this model of ALI by inducing the production of 15-epi-$LXA_4$[17]. Of note, statins can also block airway inflammation in murine models of allergic asthma[57,58,59].

### LXs in Human Airway Disease

The first identification of LXs in human tissues was in BALFs obtained from human subjects with a range of lung diseases[60]. $LXA_4$ is also present in exudative pleural effusions that are typically associated with lung or systemic inflammatory disease[61]. In mild forms of asthma, LX generation is increased in peripheral blood, induced sputum, and BALFs[14,62,63]. In contrast, multiple studies encompassing diverse ethnic backgrounds have now established that severe asthmatics display decreased LX levels, relative to subjects with mild or moderate asthma[14,62,63,64] (Table 2). Of interest, LX biosynthetic capacity is decreased in severe asthmatics[62] and the capacity for $LXA_4$ generation by whole blood is related to lung function[14,62,64], suggesting that decreased LX production may lead to a resolution defect in some individuals with severe asthma. Approximately 5–10% of adult asthmatics experience aspirin-exacerbated respiratory disease (reviewed in [65]) and LX biosynthetic capacity is also decreased in these patients[66]. Moreover, in severe asthma, ALX receptor expression is decreased in peripheral blood PMNs and EOS[63]. Levels of cysteinyl leukotrienes (CysLTs) and $LXA_4$ in both BALFs and peripheral blood demonstrate an increase in the conversion of AA to CysLTs relative to $LXA_4$ in severe compared to nonsevere asthmatics[38,63]. This change in severe asthma is related to both an increase in CysLTs and decrease in LX production[38,63]. Similarly, patients with scleroderma lung disease, marked by leukocyte infiltration and fibrosis of the lung, display enhanced $LTB_4$ and diminished $LXA_4$ levels in BALFs[67]. Cystic fibrosis is a disease of persistent lung inflammation. The nature of the airway inflammation in cystic fibrosis differs from asthma in that it is primarily related to PMN infiltration in response to chronic bacterial infection. Consistent with a theme of underproduction of these protective mediators in chronic lung inflammatory disease, decrements in LX levels are also present in cystic fibrosis[68]. Together, these data point to a role for LXs in the catabasis of human lung disease.

**TABLE 2**
**Altered Biosynthesis of Proresolving Mediators in Human Disease**

| Disease | Defect | Ref. |
|---|---|---|
| Severe asthma | Decreased $LXA_4$ in whole blood, sputum, and BALFs | [14,62,63,64] |
| | Increased CysLT to $LXA_4$ ratio in blood and BALF | [62,63] |
| Asthma exacerbation | Decreased PD1 levels in exhaled breath condensates | [97] |
| Aspirin-intolerant asthma | Decreased $LXA_4$ production from whole blood | [66] |
| Exercise-induced asthma | Decreased $LXA_4$ in plasma | [100] |
| Scleroderma lung disease | Decreased $LXA_4$ in BALFs | [67] |
| Cystic fibrosis | Decreased $LXA_4$ in BALFs | [101] |

## RESOLVINS

### Biosynthesis

Resolvins are derived from the enzymatic modification of ω-3 PUFAs and were originally identified in murine-resolving exudates[69,70]. Both eicosapentaenoic acid (EPA, C20:5) and docosahexaenoic acid (DHA, C22:6) can be converted to resolvins by murine and human tissues. The E-series resolvins are derived from EPA and are generated by a multistep process involving acetylated COX-2 or cytochrome p450 acting in concert with leukocyte 5-LO[69]. D-series resolvins, which are derived from DHA, occur in both the 17S and 17R configurations (reviewed in [11]). Biosynthesis of D-series resolvins can be catalyzed by 15-LO and 5-LO interactions, and generation of the aspirin-triggered 17R conformers proceeds via aspirin-acetylated COX-2 and 5-LO[71]. The biosynthesis of resolvins is reviewed in detail in Serhan[11] and Seki et al.[72].

### Signaling and Cellular Responses to Resolvins

The signaling pathways by which resolvins transduce anti-inflammatory and proresolving actions are rapidly evolving areas of science under active investigation. Current evidence demonstrates that resolvins signal via specific receptors. On PMNs, RvE1 acts as an antagonist and partial agonist at the $LTB_4$ receptor BLT1[73]. By blocking $LTB_4$ signaling, RvE1 decreases PMN accumulation and activation at sites of ongoing inflammation. In addition to BLT1, RvE1 can bind to the chemerin receptor ChemR23[74]. Myeloid dendritic cells express ChemR23[75] and RvE1 blocks proinflammatory responses by dendritic cells, including lipopolysaccharide-induced IL-23 release[49]. ChemR23 is also present on mucosal epithelial cells and when exposed to RvE1, CD55-dependent luminal clearance of PMNs is increased[76]. RvE1 signaling potently increases macrophage phagocytosis of apoptotic PMNs[44] and regulates PMN and T-cell expression of the chemokine receptor CCR5[77], which is an important mechanism for scavenging unwanted proinflammatory signals.

Similar to both RvE1 and LXs, more than one receptor can interact with the D-series resolvin RvD1, namely GPR32, a novel GPCR, and the ALX receptor[78]. Limited information is available for RvDs with respect to intracellular signaling. At the cellular level, RvD1 blocks PMN actin polymerization and migration toward inflammatory stimuli in a microfluidics chamber[79].

### Resolvins in Models of Lung Inflammation

RvE1 displays anti-inflammatory and proresolving properties in murine models of allergic airway inflammation, acid-induced ALI, and pneumonia[49,80,81] (Table 1). In experimental asthma, administration of RvE1 (~0.005 mg/kg) prior to aerosol allergen challenge dramatically dampens lung inflammation with decreased airway leukocytes, mucus metaplasia, and hyper-responsiveness, and significant decrements in antigen-specific IgE and IL-13 levels[49,80]. Administration of RvE1 after cessation of allergen challenge promotes resolution, as evident by more rapid decreases in airway leukocytes, airway hyper-responsiveness to inhaled methacholine, and mucus metaplasia[49]. Examination of the chemical mediators present in BALFs from these animals reveals that administration of RvE1 during the resolution phase decreased levels of the proinflammatory cytokines IL-6, IL-23, and IL-17A, thereby blocking the expansion of IL-17–generating cells, such as $T_H$-17 effector lymphocytes[49].

In addition to asthma, RvE1 proved to be anti-inflammatory and proresolving in a murine model of aspiration pneumonia in which enteric bacteria were instilled in the lung 12 h after the establishment of mild ALI[81]. Administration of RvE1 at the onset of the protocol decreases the production of proinflammatory cytokines, blocks PMN infiltration, and improves mortality[81]. RvE1 significantly decreased lung tissue levels of several proinflammatory chemokines and cytokines, including IL-1β, IL-6, HMGB-1, MIP-1α, MIP-1β, KC, and MCP-1, in a manner independent of the anti-inflammatory mediators IL-10 and $LXA_4$. In response to sterile ALI, both $LTB_4$ and KC increase in BALFs and are not significantly decreased by RvE1, despite marked decreases in lung PMNs of approximately 55%[81]. RvE1 has direct regulatory actions for PMNs that are downstream from $LTB_4$ and KC generation. For example, RvE1 can interact with BLT1 as a receptor-level antagonist[82], so functional antagonism for RvE1 at BLT1 would block $LTB_4$-mediated activation of PMNs. During experimental ALI, RvE1 inhibits PMN, but not macrophage, accumulation in the lung and protects the lung from aspiration pneumonia by increased clearance of *E. coli* infection[81]. RvE1 signals via ChemR23 on macrophages to promote the clearance of apoptotic PMNs and microbial debris[44,83], and mice deficient in ChemR23 display a proinflammatory phenotype[84]. In addition, RvE1 interacts with ChemR23 on mucosal epithelial cells to promote clearance of PMNs from apical surfaces in a CD55-dependent manner[85], and RvE1 prevents destruction of oral mucosal tissues in experimental periodontitis[86]. These findings of direct actions for RvE1 on leukocytes and mucosal epithelial cells are consistent with potent roles for this natural autacoid in regulating airway inflammation and host defense. In experimental pneumonia, the pharmacologically active dose of RvE1 was 100 ng per mouse or ~0.005 mg/kg, providing compelling evidence of this compound's potent anti-inflammatory and proresolving actions. Thus, even if only present in low amounts in lung tissues, enzymatic conversion of EPA to RvE1 would serve to limit overexuberant tissue responses to injury or infection.

The D-series resolvins also display properties consistent with a role in dampening and resolving lung inflammation. RvD1 provides protection from second-organ injury of the lung in a murine ischemia-reperfusion injury model by blocking PMN infiltration[79]. Administration of RvD2 prior to cecal ligation and puncture leads to decreased bacterial loads, cytokine levels, and neutrophil recruitment in this model of sepsis[87]. In addition, enriching mouse chow with DHA dramatically decreases the severity of an experimental model of bacterial pneumonia[88,89]. *C. elegans* ω-3 desaturase (*fat-1*) converts ω-6 PUFAs to ω-3 PUFAs, and transgenic mice expressing the *fat-1* gene experience less inflammation in ALI[88].

### Resolvins in Human Disease

DHA and EPA are found in significant amounts in fish oils and increased dietary fish ingestion is associated with health benefits. Mucosal tissues of the airway are enriched with DHA, but levels decrease in disease states, such as asthma and cystic fibrosis[90]. In humans, RvE1 is detected in the blood of subjects given EPA and can be significantly increased by ingestion of aspirin[74]. Although not a uniform finding, diets enriched with ω-3 PUFAs can have positive effects on ALI/ARDS outcomes, including decreased length of stay in intensive care units, increased oxygenation, decreased time on mechanical ventilation, and improved mortality[41,91,92]. In addition, the Physicians Health Study uncovered a correlation between increased fish intake and a lower risk of pneumonia[93].

## PROTECTINS

### Biosynthesis, Signaling, and Cellular Responses to PD1

The lead member of the protectin family is termed protectin D1 (PD1). This potent counter-regulatory lipid mediator is enzymatically derived from DHA via an epoxide-containing intermediate[94,95]. PD1 production, similar to LXs, proceeds via 15-LO[95]. PD1 binds with high affinity to human PMNs (Kd ~25 nM)[94,96], but the molecular identity of the PD1 receptor has yet to be determined. PD1 shares some proresolving counter-regulatory actions with LXs and resolvins, but utilizes distinct signaling circuits. For example, $LXA_4$, RvE1, and PD1 all inhibit PMN migration, yet neither $LXA_4$ nor RvE1 compete for PD1 binding to PMNs[95], suggesting distinct mechanisms and site of interaction.

### PD1 in Models of Lung Inflammation

Murine lungs generate PD1 and administration of exogenous PD1 (~0.0001, 0.001, and 0.01 mg/kg) decreases inflammation in a dose-dependent manner in models of allergic airway inflammation (Table 1)[97]. Administration of PD1 prior to peak airway inflammation leads to significantly less leukocyte accumulation and mucus metaplasia. Consistent with decreased lung inflammation, mice given PD1 display decreased levels of proinflammatory mediators in BALFs and decreased airway hyper-responsiveness ($ED_{200}$ for lung resistance) to inhaled methacholine[97]. Of note, PD1 decreased $LXA_4$ production *in vivo*, suggestive of independent resolution mechanisms for these two mediators. When PD1 is given as a treatment after airway inflammation is established, the compound, now present in increased amounts at an earlier time point, “jump starts” resolution and enhances the clearance of inflammatory cells (as reviewed in [11]).

### PD1 in Human Disease

PD1 is present in human lung and PD1 levels are decreased in human lung disease (Table 2)[97]. The mucosal airway is rich in DHA[90], and both PD1 and its biosynthetic precursor, 17S-hydroxy-DHA, can be detected in human exhaled breath condensates (EBCs). Comparison between healthy subjects and asthmatics experiencing an exacerbation determined that the levels of both PD1 and 17S-HDHA are decreased in EBCs during asthma exacerbations[97]. In addition, the amount of mucosal DHA is also decreased in inflammatory airway diseases, such as asthma and cystic fibrosis[90]. Thus, the properties of PD1 are consistent with protective roles in airway inflammation.

### MARESINS

Maresins are the newest family of anti-inflammatory and proresolving mediators. Maresins are 7,14 dihydroxy–containing products that are generated by activated macrophages[98]. DHA is delivered to inflamed or injured tissue by plasma exudation[79] and can be converted by macrophages to maresins to decrease the acute inflammatory response[98]. These novel proresolving compounds also block PMN trafficking and stimulate macrophage clearance of apoptotic PMNs[98]. Roles for maresins in lung biology are areas of active investigation. The presence of large numbers of alveolar macrophages in the lung and their critical role in tissue catabasis and host defense suggest important functions for maresins in the regulation of airway inflammation.

### CONCLUSIONS

There are now several lines of evidence to support fundamental roles for PUFA-derived mediators in regulating lung inflammation. In response to airway injury, infection, or noxious stimuli, the acute inflammatory response is initiated. Even at this early stage of acute inflammation, molecular signaling circuits are constructed in the airway in health for the ultimate resolution of the inflammatory response (Fig. 1). These circuits are comprised of specific mediators and receptors that transduce cell type–specific responses for anti-inflammation and resolution. The mediators are enzymatically derived from PUFAs in tightly orchestrated biosynthetic pathways that commonly involve the sequential modification of the PUFA and biosynthetic intermediates by distinct enzymes. Proresolving mediators are generated in human airways during inflammation, and defects in their production exist during severe or uncontrolled airway inflammation. When administered in animal models of inflammatory lung disease, proresolving mediators or their stable analogs display potent protective actions. Only limited information is available for intervention in human disease[99], but this genus of compounds hold promise as disease-modifying agents. With no available medical therapy to promote the resolution of asthma or ARDS, there is a substantial unmet clinical need that serves as a poignant reminder to motivate scientists to develop a more thorough understanding of the endogenous molecular circuits for resolution of airway inflammation, so that this information might be used as a window into the pathobiology of disease and as the foundation for the rational design of novel disease-remitting therapeutics.
